# Flies and Food: Grievous Dangers and How to Prevent Them

**Published:** 1917-03-31

**Authors:** 


### FLIES AND FOOD.

### Grievous Dangers and How to Prevent Them.

Dr. Cates did well to bring before the Royal Sanitary Institute his paper on the measures to be taken to prevent the contamination of food by flies. This important subject has not yet received all the attention that it deserves, either in the way of research or in the way of public measures, but already enough is known to make it certain that a very serious improvement can be made in public health by very simple measures. In the campaign against mosquitoes as the carriers of malaria and other diseases in the Tropics, one of the most interesting practical conclusions attained was that nearly every householder bred his own mosquitoes. The small accumulations of water in old tins, broken crockery, and the general rubbish of the yards near the house were the favourite resorts of these pests, and the authorities, who had the task of attempting to suppress mosquito-carried diseases, found that more immediate practical results came from domestic cleanliness than in any other way. Very much the same applies to house-flies. Occasionally there are large breeding-grounds for flies, particularly the dumps where refuse from towns is deposited and in the manure heaps from public stables—these, of course, must be dealt with by the public sanitary authorities, and there are many steps of an elaborate kind which can be taken and which ought to be taken by the public authorities. It is not very often, however, that masses of flies descend upon houses from such sources.

### How Infection is Carried.

On the other hand, every house, every cottage in town or country breeds the bulk of its own flies. At the Educational Exhibition, held by the Zoological Society of London in the Gardens during the summer of 1915, some of the most striking exhibits were directed to show this. The heads of fish, scraps of decaying fruit and vegetables, and a multitude of other objects that had been carelessly left about were shown as actual breeding-grounds of flies. The most minute care in the cleanliness of the household, of the kitchen, of all the domestic offices, and especially of refuse bins and ash bins is a vital matter. Flies lay eggs in the smallest quantities of animal or vegetable matter, and it is against these that we must look to the householder to take precautions. Fortunately, the larvæ take about four days to hatch out, so that there is a margin of time if the daily cleaning up of the rubbish and the removal of refuse should be impossible at present. The next important practical point is that the householder should learn to keep covered every kind of food and drink—sugar, jam, sweets, fruit, bread and butter, milk, beer should never be left exposed on tables or in cupboards. Flies visit everything, and immediately before alighting upon a piece of bread-and-butter they may have been visiting some filthy and contaminated spot. They carry infection in two ways—first, by particles adhering to the legs and body. A good many years ago a bacteriologist allowed a fly to walk across a gelatine plate, and subsequently it was found that each footprint grew into a colony of microbes from which the seeds of many diseases were obtained. Secondly, they have an unpleasant and dangerous habit of feeding which ought to be known. They can swallow nothing but liquid matter, and, as soon as they alight, say, upon a piece of sugar, they vomit up the contents of their stomach in a little globule upon the sugar, and then, when it has melted a little of the sugar, suck it back again. In this disgusting but effective fashion they leave behind traces from whatever place they have visited before coming to the human food.

### The Use of Traps and Baits.

The employment of methods of killing flies indoors is equally important, especially when flies first begin to appear, and every device that can be found to catch any flies at all ought to be used. It is of some importance, however, to remember tnat baits that are attractive to flies have the disadvantage of encouraging flies to come into a room. The proper time for their use, therefore, is at night, when the windows and doors are shut. In the case of ordinary bedrooms and living-rooms, scrupulous cleanliness, the use of traps and baits when the windows are shut and the rooms unoccupied ought to be sufficient, but in the case of kitchens, dining-rooms, nurseries, and, above all, sick-rooms, it is of first-rate importance that the windows should be provided with gratings of gauze or metal sufficiently large in area to allow the entrance of plenty of air, and small enough in texture to exclude flies.

The public authorities have a double task: they have to conduct operations on a large scale in refuse yards and manure dumps; they have to educate the public in what can be done by the public themselves, and in the latter task the press and the school ought to assist. "Nature study" is one of the favourite digressions of modern education; we commend the life-history of the common house-fly to all teachers of the young.

